# The prevalence of peri-implant disease following immediate implant placement and loading: a cross-sectional analysis after 2 to 10 years

**DOI:** 10.1186/s40729-020-00259-x

**Published:** 2020-10-19

**Authors:** Puria Parvini, Karina Obreja, Kathrin Becker, Maria Elisa Galarraga, Frank Schwarz, Ausra Ramanauskaite

**Affiliations:** 1 Department of Oral Surgery and Implantology, Johann Wolfgang Goethe-University, Carolinum, Frankfurt am Main, Germany; 2 Department of Orthodontics, Westdeutsche Kieferklinik, Universitätsklinikum Düsseldorf, Düsseldorf, Germany; 3 Federal University of Santa Catarina (UFSC), Florianópolis, Santa Catarina Brazil

**Keywords:** Immediate implant, Immediate load, Peri-implantitis, Peri-implant mucositis

## Abstract

**Background:**

To evaluate the prevalence of peri-implant disease after immediate implant placement and loading.

**Material and methods:**

This cross-sectional analysis included a total of 47 patients with 64 implants exhibiting a mean loading time of 2 to 10 years (4.23 ± 1.7 years). The surgical and prosthetic procedures were standardized in all patients. Peri-implant health and disease was assessed based on the established case definitions.

**Results:**

The prevalence of peri-implant health, peri-implant mucositis, and peri-implantitis amounted to 38.3%, 57.5%, and 4.2% of the patients, respectively. Mucosal recession of 1 mm was present at 4 (6%) implants. No suppuration, pain, or implant failures were reported. Ordinal logistic regression revealed that reduced keratinized mucosa height was significantly associated with the diagnosis of peri-implant mucositis and peri-implantitis (OR = 0.514, *P* = 0.0125).

**Conclusion:**

Immediate implant placement and loading was associated with high success rates at 2 to 10 years.

## Introduction

Depending on the timing of implant installation, tooth replacement with a dental implant can be performed via four approaches: immediately after tooth extraction (type 1); early, 4‑8 weeks after the extraction (type 2); in a delayed manner, 12‑16 weeks after the extraction (type 3); or conventionally, > 16 weeks following the extraction (type 4) [1, 2]. Recently, to shorten the overall treatment time, healing period, and patient morbidity, immediate implant placement and loading have become popular treatment modalities, particularly for single-implant cases. Even though recent data have pointed toward similar survival rates for immediately inserted dental implants compared to delayed and/or conventional ones, the clinical treatment outcomes (i.e., probing depth (PD) values), esthetical (pink esthetic score (PES)) treatment outcomes, and marginal bone level changes over time among the different treatment approaches remain still controversial [3–6].

Clinical and pre-clinical studies have demonstrated that immediate implant placement at the time of tooth extraction failed to prevent physiological bone remodeling, which inevitably occurs during the establishment of the peri-implant soft-tissue complex [7, 8]. The resorptive changes were accentuated on the vestibular aspect, particularly in the anterior maxilla [8, 9]. In addition, findings from one pre-clinical analysis indicated two to three times higher vertical bone resorption at immediately inserted implants than at adjacent spontaneously healed sites [10]. The aforementioned findings suggest that initial physiological bone remodeling around immediately placed implants may result in the exposure of the rough implant threads, which in turn may facilitate initial bacterial colonization.

From a clinical perspective, in the vast majority of cases, tooth extraction is deemed necessary due to persistent periapical pathology, which may be associated with altered facial alveolar bone wall morphology [11]. In fact, the integrity of the facial extraction socket wall was in turn identified as the critical factor in the decision-making process concerning the time of implant placement [2]. Nevertheless, as suggested by previous data, the presence of periapical pathology at the post-extraction site did not compromise the clinical performance of immediately inserted implants [12–14]. Residual dehiscence-type alveolar bone defects at implant sites were contrarily shown to increase the risk of peri-implant mucosal inflammation and progressive bone loss [15, 16]. Consequently, given the aforementioned, it may be hypothesized that immediately inserted implants are at a higher risk of developing peri-implant disease.

Therefore, the present analysis was intended to assess the prevalence of peri-implant tissue health or disease in immediately placed and loaded implants based on the established case definitions.

## Materials and methods

### Study design and participants

For this cross-sectional analysis, 47 partially edentulous patients (29 female and 18 male) with a total of 64 implants (Ankylos®, Dentsply Sirona Implants, Hanau, Germany) were included. The mean loading time of the implants was 4.23 ± 1.7 years (range, 2‑10 years). All patients have been treated at the Department of Oral Surgery and Implantology, Goethe University, Frankfurt, via standardized surgical and prosthetic protocols. Each patient received a detailed description of the procedure, and informed consent was obtained prior to participation. The study followed the Helsinki Declaration, as revised in 2013, and was approved by the local ethics committee (registration number: 78/18).

### Patient inclusion/exclusion criteria

The following inclusion criteria were applied for patient selection:

- Partially edentulous patients rehabilitated with immediately placed and loaded implants into the extraction sockets with periapical pathology
- Presence of keratinized mucosa > 2 mm
- Patients with treated chronic marginal periodontitis and proper periodontal maintenance care
- Non-smokers and smokers
- A good level of oral hygiene as evidenced by a plaque index (PI) < 1 at the implant level
- Attendance of yearly follow-up visits for at least 2 years

Patients were excluded for the following conditions: the presence of combined endodontic–periodontal lesions; systemic diseases that could influence the outcome of the therapy, such as diabetes (HbA1c < 7), osteoporosis, and antiresorptive therapy; a history of malignancy, radiotherapy, chemotherapy, or immunodeficiency; and pregnancy or lactation at the last follow-up.

The reason for tooth extraction in all of the cases was a persistent periapical pathology diagnosed as the presence of radiographic periapical radiolucency > 1 mm along with the clinical presence of fistula and/or pain and/or inflammatory granulation tissues in the apical region of the extraction socket.

### Surgical and prosthetic protocols

To assess the extent of periapical lesion pre-surgically, cone-beam computerized tomography (CBCT) scans were obtained for 36 patients. For the remaining patients (*n* = 11), panoramic pre-surgical radiographs were acquired.

All surgeries were performed between January 2008 and October 2017. The patients received antibiotic therapy (Amoxicillin 1‑2 g/day) for 7 days, starting 1 day before surgery. Local anesthesia (4% articaine plus epinephrine 1:100 000) was administrated before the flapless atraumatic extraction of the infected tooth using forceps and/or the Benex® (Helmut Zepf Medizintechnik GmbH, Seitingen-oberflacht, Germany) atraumatic extraction system. This was followed by a careful granulation tissue debridement and inspection of the integrity of the alveolar bone walls using a periodontal probe. Implant site preparation was performed according to the manufacturer’s surgical protocol. Bone-level platform-switch implants (Ankylos®, Dentsply Sirona Implants, Hanau, Germany) were placed 2–3 mm subcrestally along the palatal wall of the extracted socket in an optimal prosthetic position. Primary stability of ≥ 35Ncm was reached in all of the cases. Augmentation protocol was selected according to the integrity of the facial extraction socket bone wall:

- In the presence of intact extraction socket walls, the circumferential horizontal gap between the outer implant surface and the bony walls of the extraction socket was filled with a bovine-derived bone substitute (Bio-Oss spongiosa granules sized 0.25–1 mm, Geistlich, Wolhusen, Switzerland) [8]. In cases with a thin soft-tissue biotype (evaluated prior to tooth extraction based on the probe’s transparency at the mid-facial aspect; categorized as thin when the probe was visible and thick when it was not visible [17]), a connective tissue graft (CTG) harvested from the hard palate was simultaneously applied on the facial aspect via tunneling technique.
- In cases of buccal dehiscence-type defects, lateral augmentation using a bovine-derived bone substitute (Bio-Oss spongiosa granules sized 0.25–1 mm, Geistlich, Wolhusen, Switzerland) was performed [18]. For the patients additionally exhibiting a thin soft-tissue biotype, concomitant soft-tissue grafting with subepithelial CTG was performed.

**Fig. 1:**
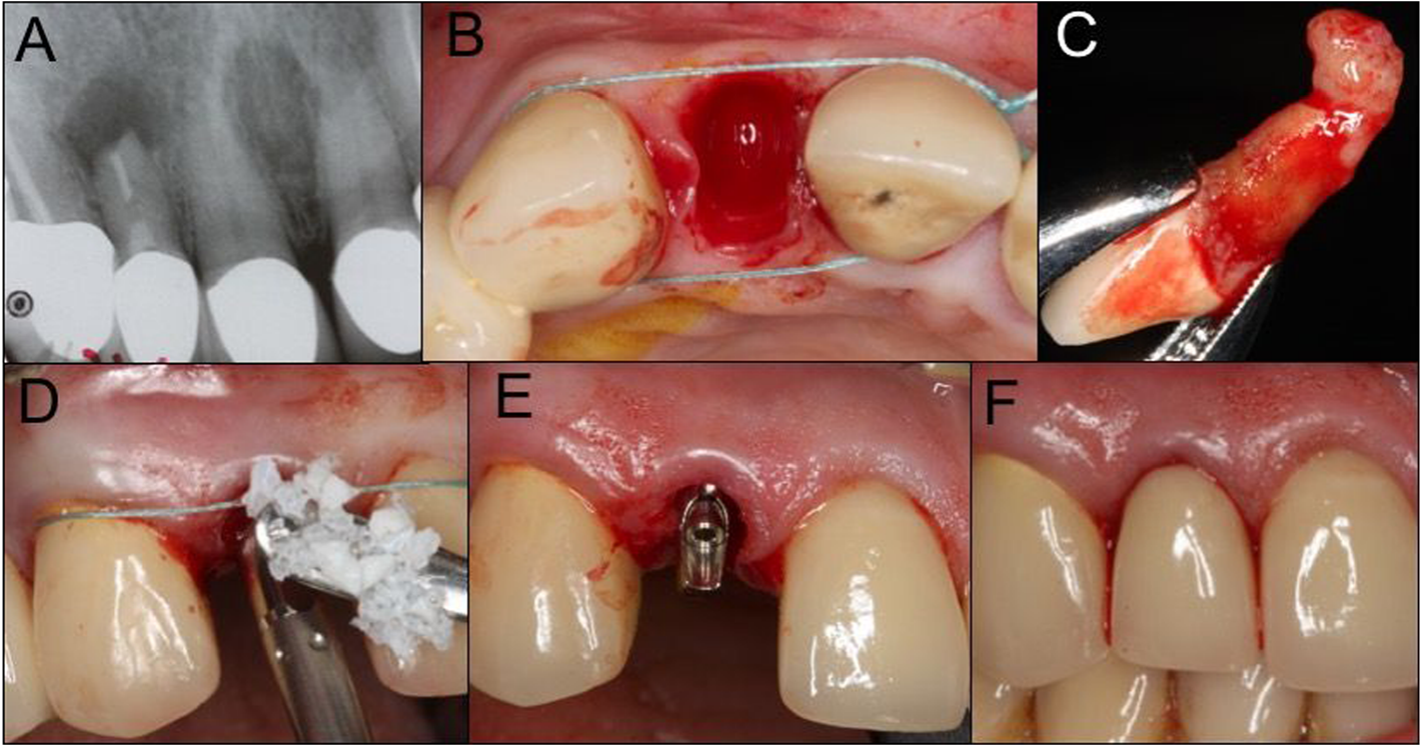
Surgical and prosthetic protocol. **a** Initial periapical radiograph showing periapical lesion tooth 12. **b** Intact post-extraction socket. **c** Extracted lateral incisor exhibiting an enucleated periapical cyst. **d** Fill of the gap between the implant and facial bone wall with xenogenic bone substitute. **e** Abutment placement. **f** Insertion of immediate restoration

Immediate provisional screw-retained abutments were inserted and acrylic resin crowns (Pro-Temp®; 3 M ESPE, GmbH, Seefeld, Germany) were fixed with temporary cement (Temp-Bond, Kerr®, GmbH, Rastatt, Germany) (Fig. 1). Occlusion was adjusted to avoid any functional loading. Definitive ceramic restorations were placed 3–4 months after the surgery [19]. Patients were enrolled in a yearly control program. All surgical and prosthetic procedures were performed by the same experienced oral surgeon and prosthodontist.

### Demographic data and implant-site characteristics

The following variables were retrieved from the patients’ files:

Patient related (Table 1): (1) age; (2) gender; (3) smoking habits (i.e., non-smoking and smoking); (4) periodontitis history; and (5) adherence to the supportive therapy.

**Table 1:** Patient demographic data

Patient demographic data	Number of patients (*n*) (%)
Patient number (*n*)	47 (100%)
Female/male (*n*)	29/18 (62%/38%)
Age (mean ± SD/median)	58.8 ± 15.5 years/58.4
Age < 60 years	25 (53.2%)
Age > 60 years	22 (46.8%)
History of periodontitis	26 (55%)
No history of periodontitis	21 (45%)
*Smoking habits*	
Smokers	11 (23%)
Non-smokers	36 (77%)
Patients adhering to the supportive therapy	40 (85%)
Frequency per year	1 time, 15 patients 2 times, 23 patients 3 times, 1 patient 4 times, 1 patient

Tooth/implant related (Table 2): (1) soft-tissue biotype; (2) integrity of the facial extraction socket bone wall (assessed intraoperatively; categorized as intact, or presenting buccal dehiscence-type defect < 30% or > 30%); (3) implant location—upper or lower jaw and anterior (i.e., canine to canine) or posterior (i.e., premolar and molar regions) segments; and (4) implant diameter and length.

**Table 2:** Tooth/implant site characteristics

Tooth/implant site characteristics	Number of teeth/implants (*n*) (%)
*Soft-tissue biotype*	
Thin	29 (45%)
Thick	36 (55%)
*Integrity of the facial alveolar bone wall following the extraction*	
Intact	50 (78%)—26 (52%) thin biotype, 24 (48% thick biotype)
Dehiscence-type buccal defect	14 (22%)—3 (21%) thin biotype, 11 (69%) thick biotype
Number of implants (*n*)	64 (100%)
*Implant location*	
Upper jaw	60 (94%)
Lower jaw	4 (6%)
Anterior region (canine-canine)	45 (70%)
Posterior region (premolar-molar)	19 (30%)
*Implant diameter*	
3.5 mm	58 (91%)
4.5 mm	6 (9%)
*Implant length*	
9.5 mm	4 (5.4%)
11 mm	17 (27.6%)
14 mm	34 (53%)
17 mm	9 (14%)

Augmentation protocol and prosthetic reconstruction (Table 3): (1) augmentation protocol (i.e., grafting of residual bone-to-implant gap or lateral augmentation with or without a concomitant subepithelial CTG); (2) type of prosthetic reconstruction (i.e., single crown or bridge); (3) cementation margin of the crown (i.e., supragingival, equigingival, or subgingival); and (4) implant loading time.

**Table 3:** Augmentation protocol and prosthetic reconstruction

Augmentation protocol and prosthetic reconstruction	Number of implants (*n*) (%)
*Augmentation protocol*	
Gap fill	24 (37.5%)
Gap fill + CTG^*^	26 (40.5%)
Lateral augmentation	11 (17%)
Lateral augmentation + CTG	3 (5%)
*Prosthetic reconstruction*	
Single-implant crown	54 (84%) implants/41 (87%) patients
Bridge reconstruction	10 (16%) implants/41 (13%) patients
*Cementation margin position*	
Supra-gingival	2 (3%)
Subgingival	45 (75%)
Equi-gingival	17 (22%)
Implant loading time	4.23 ± 1.7 years, 4.1 (median)
Loading time < 5 years	15 implants
Loading time > 5 years	49 implants

*CTG* subepithelial connective tissue graft harvested from the hard palate

### Clinical evaluation

The following clinical parameters were assessed for all patients at each implant site using a periodontal probe (PCP 12): (1) PI (Löe 1967), (2) bleeding on probing (BOP) (measured within 60 s after probing), (3) PD (measured in millimeters from the mucosal margin to the bottom of the probed pocket), (4) mucosal recession (MR) (measured in millimeters from restorative crown margin to the free mucosal margin), (5) presence or absence of suppurations (Supp), (6) mid-facial keratinized mucosa width (mid-facial KM) (measured in millimeter from the most coronal keratinized mucosa margin to the mucogingival junction on the mid-facial aspect), and (7) implant mobility (measured by manual palpation). PI, BOP, PD, and MR were assessed at six aspects around the implant: mesio-buccal, mid-buccal, disto-buccal, mesio-oral, mid-oral, and disto-oral.

### Case definitions of peri-implant diseases

Case definitions of peri-implant diseases were based on the consensus report of workgroup 4 of the 2017 World Workshop on the classification of periodontal and peri-implant diseases and conditions [20]:

1. Peri-implant tissue health defined as an absence of clinical signs of inflammation, such as BOP/Supp on gentle probing, no increase in PDs compared to previous examinations, and an absence of bone loss beyond crestal bone level changes resulting from initial bone remodeling.
2. Peri-implant mucositis defined as the presence of BOP and/or Supp on gentle probing with or without increased PDs compared to previous examinations and an absence of bone loss beyond crestal bone level changes resulting from initial bone remodeling.
3. Peri-implantitis defined as the presence of BOP and/or Supp on gentle probing, increased PDs compared to previous examination, and the presence of bone loss beyond crestal bone level changes resulting from initial bone remodeling.

When clinical signs suggested the presence of peri-implantitis, non-standardized radiographs (Heliodent Plus, Dentsply-Sirona, Bansheim, Germany) were taken using a long cone paralleling technique and compared with a baseline radiograph taken at the time of prosthesis installation [20].

### Radiographic assessment

Panoramic radiographs were obtained when clinical signs suggested peri-implant tissue inflammation. Marginal bone-level changes were assessed after comparing the baseline panoramic radiograph (i.e., taken after placing the final prosthetic reconstruction to the one retrieved at the final follow-up). Radiographs were digitized (Microtek ScanMaker i800 Plus, Hsinchu, Taiwan; LaserSoft Imaging AG, Kiel, Germany), and measurements (i.e., bone levels at baseline and at follow-up radiographs) were performed using the Sidexis XG software (Sirona Dental Systems GmbH, Bensheim, Germany). The known implant length was used to calibrate the measurement scale. Two horizontal reference lines were used—one marking the most coronal point of the peri-implant bone crest at mesial and distal sites (BC) and another tracing the implant’s most apical point (AP). Vertical lines parallel to the reference line crossing the implant’s long axis were traced perpendicularly to the BC and AP at mesial and distal sites.

### Data analysis

Two software programs (SPSS Statistics 23.0: IBM Corp., Ehningen, Germany and R (Development Core Team)) were used for the statistical analysis. Descriptive statistics were calculated for PI, BOP, PD, mid-facial KM values. The analyses were performed at both patient and implant level. Prior to the analyses at patient level, the data were pooled for the respective variables (PI, BOP, PD, mid-facial KM). When patients exhibited multiple implants with diagnoses of different severity, the worst diagnosis was selected at the patient level. When patients exhibited intact and non-intact post-extraction sockets, they were classified as intact when at least 50% or more of the sites were intact.

On the patient level, ordinal logistic regression analysis was conducted to investigate the possible effect of mean socket integrity (i.e., non-intact and intact) and mid-facial KM height on the dependent ordinal variable diagnosis (i.e., health, peri-implant mucositis, and peri-implantitis). When patients had multiple implants of which at least 50% were intact, socket integrity was classified as intact.

On implant level, a cumulative link mixed model (CLMM) fitted with adaptive Gauss-Hermite quadrature approximation with 8 quadrature points was used to assess the effect of mid-facial KM (fixed effect) on the dependent, ordinal variable diagnosis. The factor patient was used as a random effect.

On the patient level, relative risk ratios (RR) and 95% confidence intervals (95% CI) were retrieved from the intercept of the following factors: smoking status, history of periodontitis, age > 60 years.

## Results

### Demographic data and tooth/implant characteristics

Demographic data of the study population as well as tooth/implant site characteristics are presented in Tables 1 and 2. In particular, the mean age of the included patients was 58.8 ± 15.5 years, with a range from 20.6 to 84.7 years. The gender distribution revealed a higher percentage of females than males (62% and 38%, respectively). Eleven patients (23%) appeared to be smokers, and more than half of the included patients (55%) had a history of periodontitis. Majority of the patients (85%) regularly attended yearly supportive therapy visits.

With respect to the soft-tissue biotype, 45% of the tooth sites were judged to exhibit a thin biotype, whereas the remaining 55% presented with a thick soft-tissue biotype. Following tooth extraction, the majority (78%) of the extraction sockets presented with an intact alveolar bone wall on the buccal aspect. Among these sites, a similar distribution was noted for the thick and thin biotypes (48% and 52%, respectively). Dehiscence-type defects on the buccal extraction socket wall were detected in 22% cases, with the majority of the sites (69%) exhibiting a thick soft-tissue biotype. Most implants were placed in the anterior segments of the upper (73%) and lower jaws (75%). The majority of the implants (91%) had a diameter of 3.5 mm, and the most common implant length was 14 mm (53%).

### Augmentation protocol and prosthetic reconstruction

Information regarding augmentation protocol and prosthetic reconstruction are presented in Table 3. Specifically, according to the extraction socket buccal bone wall integrity and soft-tissue biotype, in the majority of the cases (40.5%), gap filling along with the CTG positioned on the buccal aspect via tunneling technique was performed. This was followed by gap filling solely (37.5%) and lateral alveolar bone augmentation with or without a CTG (5% and 11%, respectively). After 3 to 4 months, 54 implants (84%) received single crowns whereas 10 implants supported bridge reconstructions. In most of the cases (84%), the cementation margin was positioned subgingivally. Mean implant loading time was 4.23 ± 1.7 years, with a range from 2 to 10 years.

### Prevalence of peri-implant disease and clinical measurements

The prevalence of peri-implant tissue health and disease is presented in Table 4. Based on the given case definitions, the peri-implant conditions were considered to be healthy in 38.3% of the patients investigated. A total of 61.7% of the patients were diagnosed with peri-implant disease, with the majority (57.5%) exhibiting peri-implant mucositis and 4.2% of the subjects showing peri-implantitis. At the implant level, the corresponding values amounted to 48.5% (healthy peri-implant conditions), 48.5% (peri-implant mucositis), and 3% (peri-implantitis), respectively.

**Table 4:** (a) Prevalence of peri-implant tissue health and disease (i.e., peri-implant mucositis and peri-implantitis at patient and implant level; (b) clinical treatment outcomes in different patient/implant groups

**Frequency distribution of healthy and diseased sites**	**Patient level** (*n* = 47)	**Implant level** (*n* = 64)
Healthy	18 (38.3%)	31 (48.5%)
Intact extraction sockets	19 (54.3%)	26 (52%)
Non-intact extraction sockets	3 (33.3%)	5 (36%)
Peri-implant mucositis	27 (57.5%)	31 (48.5%)
Intact extraction sockets	14 (40%)	22 (44%)
Non-intact extraction sockets	6 (55.7%)	9 (64%)
Peri-implantitis	2 (4.2%)	2 (3%)
Intact extraction sockets	2 (5.7%)	2 (4%)
Non-intact extraction sockets	0	0
**Clinical and radiographic parameters**	**Patient level** (mean ± SD), median	**Implant level** (mean ± SD), median
**Plaque index**	0.22 ± 0.32, 0	0.28 ± 0.35, 0.5
Health	0.04 ± 0.11, 0	0.09 ± 0.19, 0
Peri-implant mucositis	0.38 ± 0.34, 0.167	0.43 ± 0.35, 0.33
Peri-implantitis	0.5 ± 0.71, 0.5	0.5 ± 0.71, 0.5
Intact extraction sockets	0.22 ± 0.30, 0.08	0.17 ± 0.28, 0
Non-intact extraction sockets	0.40 ± 0.41, 0.17	0.44 ± 0.42, 0.42
**Bleeding on probing (%)**	12.77 ± 18.16, 5.55%	21.09 ± 22.23, 8.33%
Health	0	0
Peri-implant mucositis	24.5% ± 12.59%, 16.67%	29.57% ± 16.5%, 25.27%
Peri-implantitis	19.44% ± 19.64%, 19.44%	25% ± 11.79%, 25.0%
Intact extraction sockets	10.87% ± 14.11%, 0%	13% ± 16.60%, 0%
Non-intact extraction sockets	18.52% ± 19.44%, 16.67%	22.61% ± 24.11%, 16.67%
**Probing depth (mm)**	2.41 ± 0.54, 2.33	2.49 ± 0.55, 2.0
Health	2.29 ± 0.52, 2.17	2.37 ± 0.51, 2.33
Peri-implant mucositis	2.59 ± 0.56, 2.67	2.59 ± 0.56, 2.67
Peri-implantitis	2.85 ± 0.33, 2.85	2.92 ± 0.59, 2.92
Intact extraction sockets	2.37 ± 0.58, 2.33	2.46 ± 0.56, 2.5
Non-intact extraction sockets	2.5 ± 0.46, 2.5	2.61 ± 0.49, 2.59
**Mid-facial KM width (mm)**	4.04 ± 1.32, 4.0	3.95 ± 1.35, 4.0
Health	4.43 ± 1.47, 4.75	4.10 ± 1.47, 4.0
Peri-implant mucositis	3.74 ± 1.09, 3.67	3.87 ± 1.23, 3
Peri-implantitis	3.25 ± 1.06, 3.25	3.0 ± 1.41, 3.0
Intact extraction sockets	3.95 ± 1.34, 4	3.84 ± 1.33, 4
Non-intact extraction sockets	4.53 ± 1.32, 4.67	4.36 ± 1.39, 4.5

With respect to the integrity of the buccal bone wall of the extraction socket, at the sites with intact extraction socket site, 5.7% of the patients presented with peri-implantitis, 40.0% with mucositis, and 54.3% with peri-implant health. The corresponding values for the non-intact sockets were 0%, 66.7%, and 33.3%. Accordingly, at the implant level, peri-implant tissue health, peri-implant mucositis, and peri-implantitis were detected at 52%, 44%, and 4% of the implants inserted into the intact extraction sockets. For the extraction sockets sites presenting with dehiscence type defects, 64% of implants were diagnosed with peri-implant mucositis, whereas the rest of the implant sites (36%) presented peri-implant tissue health.

The assessed clinical and radiological parameters are presented in Table 4. Mean PI and BOP values amounted to 0.22 and 12.77% at the patient level, and 0.28 and 21.09% at the implant level, respectively. Mean PD values amounted to 2.41 mm on patient, and 2.49 mm on implant-level data. The mean mid-facial KM width values were 4.04 mm on patient and 3.95 mm on implant level, respectively.

In total, 4 implants (6%) presented with soft-tissue recession of 1 mm: 2 of them on the mid-facial (1 implant site with thin soft-tissue biotype, 1 site with thick biotype), and the remaining 2 implants on the oral aspect (1 with thin biotype, 1 with thick biotype). All 4 implants were placed in the non-intact post-extraction sockets. None of the included implants presented suppuration. In addition, no failure or implant mobility was reported over the follow-up period.

Ordinal regression analysis revealed a significant effect of the mid-facial KM height on the diagnosis (*P* = 0.012). Specifically, patients exhibiting reduced mid-facial KM values were more frequently diagnosed with peri-implantitis/peri-implant mucositis compared to the patients exhibiting healthy implants (coefficient = −0.665; standard error = 0.266; *t* value = −2.497; odds ratio (OR), 0.5144; 95% CI, 0.293 to 0.842). The cumulative link mixed effects model revealed a non-significant maximum likelihood estimate for mid-facial KM (−0.18, error 0.1881, *z* value −0.975, *P* = 0.33). The standard deviance of the variance of the random effect patient amounted to 9.255e−05, and the condition number of the Hessian amounted to 2.1e+08 thus being larger than the threshold 10 ^ 6 for ill-defined models.

### Factors related with peri-implant tissue health/disease

None of the investigated patient-related factors (i.e., smoking, history of periodontitis, and patient age > 60 years) appeared to be associated with an increased risk for peri-implantitis or peri-implant mucositis (Table 5).

**Table 5:** Association between patient-related factors and peri-implant diseases

a) Peri-implant mucositis.			
**Peri-implant mucositis**	**Relative risk**	95% CI	*P*
Smoking	1.91	0.544‑6.721	0.302
History of periodontitis*	1.33	0.762‑2.351	0.305
Older than 60 years	1.05	0.562‑1.967	0.88
b) Peri-implantitis.			
**Peri-implantitis**	**Relative risk**	95% CI	*P*
Smoking	3.667	0.644‑20.884	0.196
History of periodontitis*	2.2	1.39‑3.48	0.148
Older than 60 years	1.10	0.256‑4.734	0.904

*Patients under maintenance

## Discussion

The present cross-sectional analysis assessed the prevalence of peri-implant tissue health and disease for immediately placed and loaded implants. According to our findings, after a follow-up period of 2 to 10 years, peri-implant tissue health was detected in 38.3% of the patients, whereas the majority of patients (61.7%) were affected by peri-implant disease. Specifically, 57.5% and 4.2% of the patients were diagnosed with peri-implant mucositis and peri-implantitis, respectively. Based on the implant-level data, peri-implant tissue health was detected as frequently as peri-implantitis, corresponding to 48.5% and 48.5% of the implants. In agreement with patient-level estimation, peri-implantitis was not a common finding, and detected in 3% of the implants.

Few previous studies have attempted to elucidate the occurrence of biological complications related to immediate implant placement. In particular, over a period of 1 to 9 years, 18% to 30% of the implants were affected by peri-implant mucositis, and 9% of the implants presented with the clinical signs of peri-implantitis (defined as the presence of BOP + PD ≥ 4 mm + significant bone loss) [6, 21]. The comparison of the prevalence of peri-implant mucositis in delayed and immediate implants showed similar outcomes (delayed: 7/34 (21%) of the implants; immediate: 6/34 (18%)), whereas immediate implants were more frequently affected by peri-implantitis (immediate: 3/43 (9%); delayed: 1/34 (3%)) [21]. A noteworthy finding indicates that the prevalence of peri-implant mucositis observed in the present analysis was twice as high. The latter discrepancy could be at least partially explained by the different definitions applied to the disease, as well as by the patient sample enrolled in the current study, which was twice as large.

The integrity of the facial extraction socket wall was reported to be the critical factor in the decision-making process regarding the time of implant placement [2]. Inferior clinical, radiographic, and patient-reported outcomes have been obtained following immediate implant placement at sites with compromised alveolar sockets [22]. Concerning the integrity of the extraction socket’s buccal wall, peri-implant tissue was more frequently healthy in patients exhibiting implants inserted into the intact extraction sockets than in those with dehiscence-type defects on the buccal aspect (54.3% vs. 33.3% of the implants, respectively). Conversely, peri-implant mucositis prevalence was higher for implant sites with buccal dehiscence defects (66.7% vs. 40% of the implants). The two implants diagnosed with peri-implantitis were, however, installed in intact extraction sockets. As previous clinical data clearly demonstrated that residual defects around the implant of ≥ 1 mm in height increase the risk of developing peri-implant disease, in the present study, all implant sites presenting with dehiscence defects were laterally grafted, using xenogeneic filler material [15, 16]. In fact, consistent with our findings, a recent analysis reported a similar incidence of peri-implant mucositis and peri-implantitis between the implant sites treated with and without lateral hard tissue grafting pointing toward the safety of the procedure (grafted sites, 68% and 5%; non-grafted sites, 61% and 10%, respectively) [23].

In the present analysis, the reason for tooth extraction in all of the cases was persistent periapical pathology, which is, in general, one of the major reasons for tooth extraction. Periapical tissue pathology at the tooth sites was shown to correlate with reduced facial alveolar thickness [24]. Nevertheless, as suggested in the previous comparative clinical investigations, implant placement into post-extraction sites with periapical pathology did not compromise implant survival rates, or clinical and radiographic outcomes when compared to immediate implant insertion into healthy post-extraction sockets [12–14]. However, it must be further considered that implant placement into infected sites may increase the risk of retrograde peri-implantitis (i.e., periapical inflammation) [25]. In the present study, the implant survival rate amounted to 100% over the investigation period, and none of the implants developed symptoms related to the prior condition (i.e., pain, mobility, fistula, or swelling), which corroborates the results of previous clinical studies [12–14].

Previous clinical data suggested that immediate implant placement was commonly associated with a high frequency of mid-facial soft-tissue recession (9% to 41% of sites after 1- to 3-year follow-ups) [26]. Moreover, a thin soft-tissue biotype was found to be one of the major factors related to soft-tissue recession for the immediately placed implants [27–29]. Therefore, as a preventive measure, in cases involving a thin soft-tissue biotype, the use of a CTG has been proposed [30, 31]. In the present investigation, soft-tissue recession of 1 mm was not a common finding, detected in four 4 (6%) implants, with an equal distribution between the thick and thin soft-tissue biotypes.

The findings of the present analysis pointed to a significant association between the reduced mid-facial KM width and peri-implant disease (i.e., peri-implant mucositis and peri-implantitis). This observation aligns with the results of previous clinical investigations that reported a higher risk for showing signs of peri-implantitis (OR of 1.9 and 3.89) for implants with the absence or < 1 mm of KM [32–34]. On the other hand, the role of KM width in maintaining peri-implant tissue health remains controversial, since one recent clinical assessment of 87 compliant patients over a 5-year follow-up period revealed no correlation between mid-facial KM width around dental implants and parameters related to peri-implant diseases [35].

In corroboration to the previous clinical analyses, none of the investigated patient-related variables (i.e., patient age > 60 years, smoking, and history of periodontitis) were found to be associated with neither peri-implant mucositis nor peri-implantitis [32, 36, 37]. Nevertheless, the opposing cross-sectional data pointed toward a significant association between the smoking habit and diagnosis of peri-implantitis [37]. With respect to the patients’ history of periodontitis, the results of the present analysis should be interpreted with caution, as the study was based on a relatively small patient subset with a history of periodontitis, all of which were enrolled in a regular maintenance program.

The present analysis included clinical cases where either the grafting of a bone-to-implant gap or dehiscence-type defect was performed along with or without simultaneous subepithelial CTG placed on the facial aspect. It should be mentioned, however, that due to a limited number of patients enrolled in different treatment groups, no subgroup analysis was conducted to evaluate the extent augmentation protocol may possibly have influenced the results of the present analysis. Furthermore, a total of 10 patients exhibited more than one implant. Whenever only a few patients exhibit multiple measurements, statistical analyses are challenging. Generalized mixed models or CLMM estimates can be unstable if there is a small number of observations within clusters or if there are very few clusters from which within-group correlation is estimated. In the present analysis, the estimated within-group variance was close to zero, as the majority of data had zero variance (only one measurement per patient available). Therefore, the condition number of the Hessian, measuring the empirical identifiability of the model, indicated that the model was not well defined. Therefore, data were aggregated on patient level to achieve a more robust analysis. This analysis, however, has the limitation that the correlation of the multiple measurements from ten patients could not be modeled.

In conclusion, and within its limitations, the current analysis indicated that immediate implant placement and loading at infected sites were associated with high success rates at 2 to 10 years. Further randomized, controlled clinical trials elaborating on the long-term clinical performance of dental implants immediately placed and loaded are needed.

## Data Availability

Not applicable.
